# Occupational Fatigue and Multidimensional Traffic Risk Outcomes Among Motorcycle-Based Food Delivery Workers: Cross-Sectional Study

**DOI:** 10.2196/92667

**Published:** 2026-06-12

**Authors:** Sookyung Kim, Chang Gi Park, Mi-So Shim

**Affiliations:** 1 School of Nursing, Soonchunhyang University, Cheonan, Republic of Korea; 2 Department of Population Health Nursing, College of Nursing, University of Illinois Chicago, Chicago, IL, United States; 3 College of Nursing, Keimyung University, 1095, Dalgubeol-daero, Dalseo-gu, Daegu, Republic of Korea, 82 532587668, 82 532587616

**Keywords:** accidents, traffic, fatigue, occupational health, motorcycles, health risk behavior

## Abstract

**Background:**

Although motorcycle-based food delivery workers face a significant risk of accidents, previous research has primarily focused on traffic accidents, neglecting the multidimensional nature of safety, which includes perceived accident risk, near-miss experiences, and accident-related anxiety.

**Objective:**

This study addresses this gap by investigating how occupational fatigue and health behaviors are associated with the Traffic Accident Risk Index (TARI) and its subdomains (near-miss experiences, self-rated accident anxiety, and other-rated accident anxiety).

**Methods:**

A cross-sectional study was conducted. Data were collected from South Korean delivery workers via an online survey and analyzed using multiple linear regression.

**Results:**

In total, 336 workers were included in the analysis. Occupational fatigue was positively associated with the overall risk index (*B*=0.017, 95% CI 0.012 to 0.021; *P*<.001) and all subdomains, including near-miss experiences (*B*=0.016, 95% CI 0.011 to 0.021; *P*<.001), other-rated accident anxiety (*B*=0.019, 95% CI 0.013 to 0.024; *P*<.001), and self-rated accident anxiety (*B*=0.016, 95% CI 0.011 to 0.021; *P*<.001). Inconsistent helmet use was associated with a higher TARI (*B*=0.413, 95% CI 0.030 to 0.796; *P*=.04), other-rated accident anxiety (*B*=0.495, 95% CI 0.025 to 0.966; *P*=.04), and self-rated accident anxiety (*B*=0.493, 95% CI 0.036 to 0.949; *P*=.04). Insufficient physical activity was associated with a higher TARI (*B*=0.368, 95% CI 0.016 to 0.719; *P*=.04) and self-rated accident anxiety (*B*=0.586, 95% CI 0.168 to 1.005; *P*=.006). Current smoking was associated with near-miss experiences (*B*=0.298, 95% CI 0.041 to 0.555; *P*=.02). Conversely, shorter break times were associated with lower accident risk (30 min to 1 h: *B*=−0.329, 95% CI −0.650 to −0.007; *P*=.045) and near-miss experiences (<30 min: *B*=−0.474, 95% CI −0.936 to −0.011; *P*=.045; 30 min to 1 h: *B*=−0.470, 95% CI −0.846 to −0.095; *P*=.01) than breaks exceeding 2 hours.

**Conclusions:**

Occupational fatigue was associated with a higher overall perceived accident risk, more near-miss experiences, and greater accident-related anxiety. Modifiable health behaviors showed additional domain-specific associations. Prevention efforts may benefit from combining fatigue management with strategies to improve helmet use, increase physical activity, and support smoking cessation. Future research should refine the measurement of break time and establish evidence-based rest guidance for motorcycle-based delivery workers.

## Introduction

In recent years, the number of gig workers, defined as individuals who provide labor through digital platforms, has increased significantly worldwide [1]. The rapid expansion of food delivery applications has markedly increased the number of motorcycle-based food delivery workers (MFDWs) [2]. Because MFDWs deliver orders using motorcycles, they are routinely exposed to road hazards and face a disproportionately high risk of traffic accidents when compared with other occupational groups [3]. Furthermore, algorithmic management and piece-rate payment systems often compel MFDWs to operate under intense time pressure, potentially shaping risky riding behaviors and elevating crash risks [4]. Moreover, the concept of rest in the gig economy is often ambiguous; periods labeled as breaks may conflate restorative rest with unpaid waiting times for algorithmically assigned tasks, potentially hindering effective recovery [5].

Although traffic accidents involving physical injury remain a primary concern [6], safety outcomes in this workforce encompass more than just accidents. Near-miss experiences and accident-related anxiety are critical yet understudied dimensions of traffic safety. Accident-related anxiety reflects not only a psychological response to danger but also a perceived vulnerability to crashes [7]. Accident-related anxiety, when combined with occupational fatigue, a state of exhaustion known to impair cognitive performance and elevate anxiety, may further compromise safety [8]. Importantly, from a preventative perspective, identifying early indicators before actual crash events is crucial for recognizing individuals at high risk of traffic accidents. Measures such as near-miss experiences and accident-related anxiety may provide valuable insight into preaccident risk and support the development of preventive interventions [9]. Therefore, understanding traffic safety requires a multidimensional approach that considers near-miss experiences and anxiety along with actual accidents among MFDWs.

Previous reviews have consistently reported sociodemographic characteristics and behavioral factors as key risk factors for adverse traffic safety outcomes among motorcycle riders. Sociodemographic factors, such as younger age and lower education level, along with behavioral characteristics, including alcohol consumption, smoking, nonhelmet use, and risky driving behaviors, have been consistently associated with traffic crashes and injuries [10,11]. Additionally, occupational status–related factors, such as long working hours and work schedules, are important correlates of traffic safety outcomes, and rider fatigue has been proposed as a significant risk factor [12]. Occupational fatigue has been identified as an important risk factor among motorcycle riders; however, it has rarely been assessed using validated instruments. Moreover, comprehensive analyses that simultaneously consider occupational fatigue, health-related behaviors, occupational status, and sociodemographic characteristics with adequate control for potential confounders remain limited.

Despite a growing literature on gig delivery workers, key gaps remain. Existing evidence has largely relied on crash- or injury-related outcomes, whereas near-miss or traffic-conflict events, widely discussed as proactive surrogate safety indicators, have been less commonly examined in this population [6,13]. Furthermore, the psychological aspects of safety, particularly traffic accident anxiety, have seldom been analyzed in relation to occupational fatigue and health behaviors. Additionally, existing studies lack comprehensive analyses that integrate occupational fatigue measured with validated instruments alongside health-related behaviors, occupational status, and sociodemographic characteristics, while adequately controlling for potential confounders. Addressing these gaps is vital to formulate prevention strategies that transcend regulatory enforcement. Therefore, this study aimed to bridge these limitations by investigating the work- and health-related determinants of traffic safety among MFDWs. Using a multidimensional framework, we assessed 3 distinct domains: Traffic Accident Risk Index (TARI), near-miss experiences, and accident-related anxiety. By integrating these experiential and psychological dimensions, this study aimed to provide comprehensive evidence to help occupational health policymakers improve the safety and well-being of platform delivery workers.

This study aimed to (1) assess the levels of traffic accident risk, including near-miss experiences, other-rated accident anxiety, self-rated accident anxiety, and occupational fatigue; (2) describe the demographic characteristics, occupational status, and health behaviors of MFDWs; (3) investigate the relationship between TARI and occupational fatigue; and (4) identify factors associated with TARI and its 3 subdomains among MFDWs.

## Methods

### Study Design

A cross-sectional descriptive study design was used. This study was reported in accordance with the STROBE (Strengthening the Reporting of Observational Studies in Epidemiology) guidelines (Checklist 1).

### Participants

The participants were MFDWs nationwide who were registered with platform-based delivery services and were recruited using convenience sampling. Given the fragmented nature of platform labor and the absence of a centralized registry for gig workers, MFDWs are difficult to reach through probability-based recruitment. Consequently, this sampling strategy was adopted to maximize access to actively working riders in real-world settings. Inclusion criteria were (1) being an adult and (2) having worked as an MFDW for more than 3 months. Individuals who could not understand or communicate in Korean were excluded.

### Data Collection

The participants were recruited between August and October 2023 through offline and online channels. For offline recruitment, the researcher contacted multiple delivery agencies in 2 cities (Asan and Cheonan, Chungcheongnam-do, South Korea) and posted study notices at agencies that agreed to participate. MFDWs who expressed interest were directed to the study information sheet and consent form using QR codes. For online recruitment, a professional survey agency was contracted to distribute study invitations to MFDWs registered on major platform-based delivery services in South Korea. Participants began the survey after confirming their consent online. The study information sheet and informed consent form included details such as the right to withdraw from the study at any time during the study period; guarantees of anonymity; benefits, risks, inconveniences associated with participation; and confidentiality of personal information and records. In total, 336 participants voluntarily submitted consent forms and online survey responses.

### Measures

#### Demographic Characteristics

Demographic characteristics were assessed using a questionnaire on age, gender, marital status, education, and socioeconomic status. Educational level was assessed based on the participants’ highest degree and categorized into 3 groups: middle school or below, high school, and college or above. Socioeconomic status was classified as high, moderate, or low.

#### Occupational Status

Occupational status was assessed using items related to career year, weekly working hours, breaks (including mealtimes), and helmet use. Career year was assessed by identifying the total duration that the participants had worked as professional riders. Break times, including mealtimes, were measured by asking the participants to report their daily break duration: 30 minutes or less, 30 minutes to less than 1 hour, 1 to less than 2 hours, or 2 hours or more. Finally, helmet use was assessed by asking the participants whether they always wear, usually wear, usually do not wear, or never wear. The responses were dichotomized into consistent helmet use (always or usually wear it) and inconsistent helmet use (usually do not wear it or never wear it).

#### Health-Related Characteristics

The following health-related characteristics were assessed: BMI, smoking status, alcohol problems, physical activity level, sleep duration, and the presence of disease. BMI was calculated from participants’ self-reported height and weight by dividing weight (kg) by the square of height (m^2^). The participants were then classified as underweight (<18.5 kg/m^2^), normal (18.5 to <23.0 kg/m^2^), overweight (23.0 to <25.0 kg/m^2^), or obese (≥25.0 kg/m^2^). Smoking status was assessed by asking whether the participant currently smoked (“yes” or “no”). Alcohol problems were assessed using the Alcohol Use Disorder Identification Test Concise, which consists of 3 items [14]. The participants were classified as having an alcohol problem if their total score was 6 or higher for males and 5 or higher for females [14]. Physical activity level was measured by asking the participants how many times they exercised per week. Sleep duration was classified into 7 to 9 hours and less than 7 or more than 9 hours based on adult recommendations and increased crash risk among drivers [15,16]. The presence of a disease was determined by asking a physician whether the participant had been diagnosed with any medical condition.

#### Occupational Fatigue

Fatigue was assessed using the Korean version of the Swedish Occupational Fatigue Inventory [17], originally developed by Ahsberg [18]. It consists of 20 items divided into 5 subscales (lack of energy, physical exertion, physical discomfort, lack of motivation, and sleepiness), each of which is scored on a 7-point Likert scale from 0 (“strongly disagree”) to 6 (“strongly agree”). The sum of all the items ranged from 0 to 120 points, with a higher score indicating more severe fatigue. Cronbach α was in the range from 0.81 to 0.92 (lack of energy=0.92, physical exertion=0.87, physical discomfort=0.81, lack of motivation=0.92, and sleepiness=0.89) at development and was 0.95 in this study.

#### Traffic Accident Risk Index

Developed by Lee and Lee [9] and revised by Song and Lee [19], TARI captures perceived and self-reported dimensions of traffic risk, including near-miss experiences and accident-related anxiety, rather than objective accident incidence. The questionnaire was divided into 3 subdomains: 2 items assessed near-miss experiences as either perpetrators or victims, 2 items assessed other-rated accident anxiety (ie, the perceived likelihood of being involved in an accident as judged by people around the participant), and 2 items assessed self-rated accident anxiety. Each item was measured on a 5-point Likert scale ranging from 1 (“not at all”) to 5 (“very much”). The overall score was calculated by averaging the scores of all items (range 1‐5), with higher scores indicating a higher likelihood of traffic accidents. The score was treated as a continuous variable, and multiple linear regression was applied, as this approach is commonly used and appropriate for composite measures derived from Likert-type items. Cronbach α was 0.87 at development and 0.88 in this study. For the subdomains, Cronbach α values were 0.75 for near-miss experiences, 0.84 for other-rated accident anxiety, and 0.87 for self-rated accident anxiety.

### Data Analysis

Data were analyzed using Stata software (version 18.0; StataCorp LP). The TARI and occupational fatigue levels, along with the participants’ demographic characteristics, occupational status, and health-related characteristics, were analyzed using frequencies, percentages, means, and SDs. Correlations between occupational fatigue, TARI, and its domains were analyzed using Pearson correlation coefficient. Multiple regression analyses were conducted separately for TARI and each of its 3 domains to identify associated risk factors. To assess the potential for common method bias, Harman single-factor test was conducted using 26 items from the 2 primary psychological constructs (occupational fatigue and TARI).

### Ethical Considerations

This study was approved by the Institutional Review Board of Soonchunhyang University (202305-SB-065) in accordance with the Declaration of Helsinki. Informed consent was obtained from all participants prior to their participation in the survey. All collected data were anonymized and deidentified to protect participants’ privacy and confidentiality. Participants received a coupon worth KRW 10,000 (≈US $7) as a token of appreciation.

## Results

### General Characteristics

General characteristics of the participants are shown in Table 1. Approximately 90.5% (304/336) of the participants were male, with a mean age of 30.78 (SD 6.30) years. Among them, 77.7% (261/336) were single, 16.4% (55/336) had graduated from middle school, and 5.4% (18/336) reported a high socioeconomic status. Regarding occupational status, 9.8% (33/336) had less than 1 year of career experience; 29.2% (98/336) worked less than 40 hours per week; 25.3% (85/336) had breaks of less than 30 minutes, including mealtimes; and 93.2% (313/336) reported consistent helmet use (always or usually wearing a helmet). In terms of health-related characteristics, 47.0% (158/336) had a normal BMI, 36.9% (124/336) were smokers, 26.5% (89/336) reported alcohol problems, 10.4% (35/336) engaged in physical activity almost every day, the mean sleep duration was 7.79 (SD 1.77) hours, 37.2% (125/336) reported short or long sleep duration, and 14.6% (49/336) reported the presence of disease.

**Table 1.** General characteristics of motorcycle-based food delivery workers (N=336).

Variables and characteristics	Values
Demographics	
Age (y)	
Mean (SD)	30.78 (6.30)
18‐29, n (%)	172 (51.7)
30‐39, n (%)	130 (39.0)
≥40, n (%)	31 (9.3)
Gender, n (%)	
Female	32 (9.5)
Male	304 (90.5)
Marital status, n (%)	
Single	261 (77.7)
Married	63 (18.7)
Divorced/widowed	12 (3.6)
Education, n (%)	
≤Middle school	55 (16.4)
High school	177 (52.7)
≥College	104 (30.9)
Socioeconomic status, n (%)	
High	18 (5.4)
Moderate	184 (54.7)
Low	134 (39.9)
Occupational status	
Career year, n (%)	
≤1	33 (9.8)
>1 to 3	166 (49.6)
>3 to 5	110 (32.8)
≥5	26 (7.8)
Working hours per week	
Mean (SD)	45.91 (15.81)
<40, n (%)	98 (29.2)
40‐52, n (%)	138 (41.6)
>52, n (%)	96 (28.9)
Breaks including mealtimes, n (%)	
<30 min	85 (25.3)
30 min to 1 h	131 (39.0)
1 to 2 h	82 (24.4)
≥2 h	38 (11.3)
Helmet use, n (%)	
Consistent	313 (93.2)
Inconsistent	23 (6.9)
Health-related characteristics	
BMI	
Mean (SD)	23.35 (3.01)
Normal, n (%)	158 (47.0)
Underweight, n (%)	11 (3.3)
Overweight, n (%)	97 (29.0)
Obese, n (%)	69 (20.6)
Smoking status, n (%)	
Current smoker	124 (36.9)
Nonsmoker	212 (63.1)
Alcohol problems, n (%)	
Yes	89 (26.5)
No	247 (73.5)
Physical activity, n (%)	
Almost every day	35 (10.4)
3‐4 times per week	69 (20.5)
1‐2 times per week	91 (27.1)
Almost never	141 (42.0)
Sleep duration	
Mean (SD)	7.79 (1.77)
<7 or >9, n (%)	125 (37.2)
7‐9, n (%)	198 (61.3)
Presence of disease, n (%)	
Yes	49 (14.6)
No	287 (85.4)

### Traffic Accident Risk and Occupational Fatigue Levels

The average TARI score was 2.65 (SD 0.97), with scores ranging from 1 to 5. The means and SDs of the 3 domains of TARI (near-miss experiences, other-rated accident anxiety, and self-rated accident anxiety) were 3.03 (SD 1.08), 2.56 (SD 1.21), and 2.38 (SD 1.11), respectively. The mean total score for occupational fatigue was 48.46 (SD 26.58), with scores ranging from 0 to 113. Table 2 also presents the mean scores for the 5 occupational fatigue subscales.

**Table 2.** Characteristics of the occupational fatigue and the Traffic Accident Risk Index among motorcycle-based food delivery workers (N=336).

Variables	Mean (SD; range^a^)
Occupational fatigue	48.46 (26.58; 0-113)
Lack of energy	10.91 (5.93; 0-24)
Physical exertion	7.91 (5.87; 0-20)
Physical discomfort	9.74 (6.15; 0-24)
Lack of motivation	9.01 (5.59; 0-24)
Sleepiness	10.89 (5.85; 0-24)
Traffic Accident Risk Index	2.65 (0.97; 1-5)
Near-miss experiences	3.03 (1.08;1-5)
Other-rated accident anxiety	2.56 (1.21; 1-5)
Self-rated accident anxiety	2.38 (1.11;1-5)

a Minimum-maximum.

Exploratory univariate comparisons suggested that TARI and subdomain scores varied across several demographic, occupational, and health-related characteristics (Multimedia Appendix 1). These results were used to contextualize and inform the multivariable models.

### Correlation Between Traffic Accident Risk Outcomes and Occupational Fatigue

**Figure 1.**
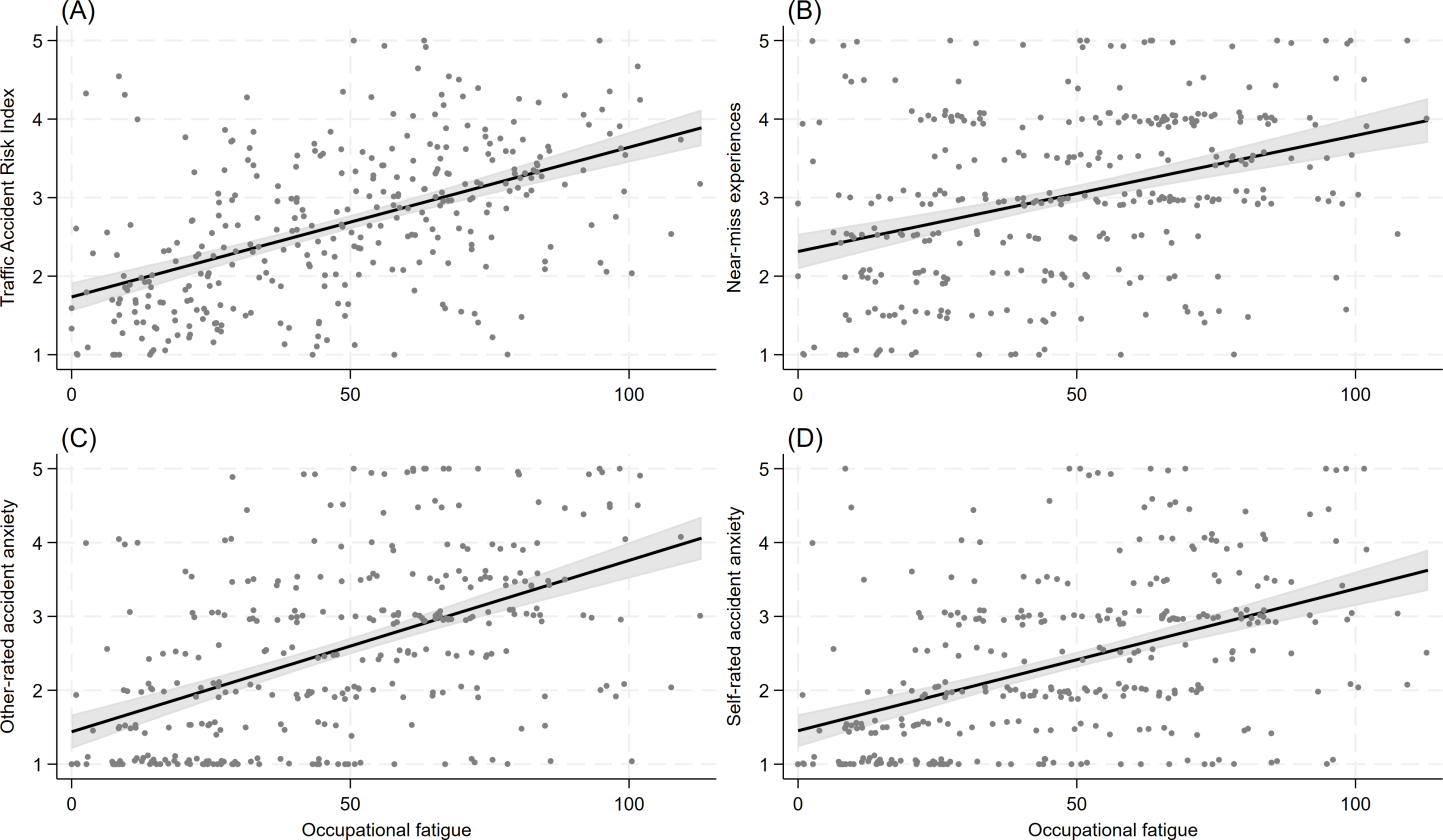
Unadjusted associations between occupational fatigue and traffic accident risk outcomes. Scatterplots show bivariate relationships between occupational fatigue and (A) Traffic Accident Risk Index, (B) near-miss experiences, (C) other-rated accident anxiety, and (D) self-rated accident anxiety. Solid lines indicate fitted values from linear regression, and shaded bands represent 95% CIs. Multivariable-adjusted estimates are reported in Table 3.

Occupational fatigue was positively correlated with the overall TARI and its subdomains, including near-miss experiences and accident-related anxiety (*r*=0.36-0.52, all *P*<.001; Figure 1). These unadjusted patterns were consistent with the multivariable models (Table 3). The full correlation matrix is provided in Multimedia Appendix 2.

**Table 3.** Factors affecting Traffic Accident Risk Index and its 3 domains among motorcycle-based food delivery workers (N=336).

Variables	TARI^a^		Near-miss experiences		Other-rated accident anxiety		Self-rated accident anxiety	
	*B* (SE)	95% CI	*B* (SE)	95% CI	*B* (SE)	95% CI	*B* (SE)	95% CI
Age (y) (ref^b^: 18‐29 y)								
30‐39	0.119 (0.114)	−0.104 to 0.343	0.148 (0.133)	−0.113 to 0.409	0.053 (0.139)	−0.220 to 0.327	0.157 (0.135)	−0.109 to 0.423
≥40	0.208 (0.217)	−0.219 to 0.635	0.349 (0.254)	-0.150 to 0.849	0.215 (0.266)	−0.309 to 0.738	0.060 (0.258)	−0.448 to 0.569
Sex (ref: female)								
Male	0.155 (0.172)	−0.184 to 0.493	−0.081 (0.201)	−0.477 to 0.315	0.353 (0.211)	−0.062 to 0.769	0.192 (0.205)	−0.212 to 0.595
Marital status (ref: single)								
Married	0.022 (0.148)	−0.269 to 0.314	−0.030 (0.173)	−0.371 to 0.311	−0.085 (0.182)	−0.442 to 0.273	0.181 (0.177)	−0.167 to 0.529
Widowed/divorced	−0.362 (0.330)	−1.011 to 0.288	−0.348 (0.386)	−1.107 to 0.411	−0.458 (0.405)	−1.254 to 0.339	−0.280 (0.393)	−1.054 to 0.494
Education (ref: ≥College)								
≤Middle school	0.209 (0.203)	−0.190 to 0.608	−0.287 (0.237)	−0.753 to 0.180	0.525^c^ (0.249)	0.036 to 1.014	0.389 (0.242)	−0.086 to 0.865
High school	−0.028 (0.106)	−0.237 to 0.181	−0.239 (0.124)	−0.484 to 0.006	0.012 (0.130)	−0.245 to 0.268	0.143 (0.127)	−0.107 to 0.392
SES^d^ (ref: High)								
Moderate	−0.049 (0.246)	−0.533 to 0.436	-0.353 (0.288)	−0.919 to 0.213	−0.085 (0.302)	−0.679 to 0.509	0.292 (0.293)	−0.285 to 0.869
Low	−0.005 (0.244)	−0.486 to 0.476	−0.253 (0.286)	−0.815 to 0.310	−0.066 (0.300)	−0.656 to 0.523	0.303 (0.291)	−0.270 to 0.876
Career year (ref: ≤1 y)								
>1 to 3	0.012 (0.173)	−0.328 to 0.352	0.104 (0.202)	−0.293 to 0.502	−0.006 (0.212)	−0.423 to 0.411	−0.062 (0.206)	−0.467 to 0.343
>3 to 5	0.154 (0.187)	−0.214 to 0.522	0.189 (0.218)	−0.241 to 0.619	0.252 (0.229)	−0.199 to 0.703	0.022 (0.223)	−0.416 to 0.460
≥5	−0.119 (0.239)	−0.590 to 0.352	0.040 (0.280)	−0.511 to 0.591	−0.269 (0.294)	−0.847 to 0.309	−0.128 (0.285)	−0.689 to 0.434
Working hours per week (ref: <40 h)								
40‐52	−0.202 (0.141)	−0.479 to 0.075	−0.300 (0.164)	−0.623 to 0.024	0.001 (0.172)	−0.339 to 0.340	−0.308 (0.168)	−0.637 to 0.022
>52	−0.078 (0.150)	−0.373 to 0.218	−0.044 (0.176)	−0.390 to 0.301	0.108 (0.184)	−0.255 to 0.471	−0.297 (0.179)	−0.649 to 0.056
Break time (ref: ≥2 h)								
≤30 min	−0.281 (0.201)	−0.677 to 0.115	−0.474^c^ (0.235)	−0.936 to −0.011	−0.228 (0.247)	−0.714 to 0.257	−0.141 (0.240)	−0.613 to 0.331
30 min to 1 h	−0.329^c^ (0.163)	−0.650 to −0.007	−0.470^c^ (0.191)	−0.846 to −0.095	−0.317 (0.200)	−0.711 to 0.077	−0.198 (0.194)	−0.581 to 0.184
1 to 2 h	−0.237 (0.175)	−0.581 to 0.108	−0.254 (0.205)	−0.657 to 0.149	−0.211 (0.215)	−0.634 to 0.212	−0.245 (0.209)	−0.656 to 0.165
Helmet use (ref: consistent)								
Inconsistent	0.413^c^ (0.195)	0.030 to 0.796	0.251 (0.228)	−0.197 to 0.699	0.495^c^ (0.239)	0.025 to 0.966	0.493^c^ (0.232)	0.036 to 0.949
BMI (ref: underweight to normal)								
Overweight/Obese	–0.096 (0.105)	−0.302 to 0.110	0.009 (0.122)	−0.232 to 0.249	−0.135 (0.128)	−0.387 to 0.118	−0.162 (0.125)	−0.407 to 0.083
Smoking (ref: nonsmoker)								
Current smoker	0.008 (0.112)	−0.212 to 0.227	0.298^c^ (0.130)	0.041 to 0.555	−0.172 (0.137)	−0.441 to 0.098	−0.104 (0.133)	−0.366 to 0.158
Alcohol problem (ref: no)								
Yes	0.135 (0.110)	−0.082 to 0.352	0.170 (0.129)	−0.084 to 0.424	0.085 (0.135)	−0.182 to 0.351	0.151 (0.131)	−0.108 to 0.410
Physical activity (ref: almost everyday)								
3‐4 times/wk	0.246 (0.199)	−0.145 to 0.637	0.222 (0.232)	−0.235 to 0.679	0.160 (0.244)	−0.320 to 0.639	0.356 (0.237)	−0.110 to 0.822
1‐2 times/wk	0.247 (0.189)	−0.124 to 0.618	0.336 (0.221)	−0.098 to 0.770	0.064 (0.231)	−0.391 to 0.520	0.340 (0.225)	−0.102 to 0.782
Almost never	0.368^c^ (0.179)	0.016 to 0.719	0.340 (0.209)	−0.071 to 0.751	0.177 (0.219)	−0.254 to 0.607	0.586^e^ (0.213)	0.168 to 1.005
Sleep duration (ref: 7‐9 h)								
<7 or >9 h	−0.028 (0.099)	−0.223 to 0.167	0.017 (0.116)	−0.211 to 0.245	–0.096 (0.122)	−0.335 to 0.143	−0.006 (0.118)	−0.239 to 0.226
Presence of disease (ref: no)								
Yes	−0.017 (0.149)	−0.310 to 0.275	0.100 (0.174)	−0.242 to 0.442	–0.001 (0.182)	−0.360 to 0.358	−0.151 (0.177)	−0.500 to 0.198
Occupational fatigue	0.017^f^ (0.002)	0.012 to 0.021	0.016^f^ (0.003)	0.011 to 0.021	0.019^f^ (0.003)	0.013 to 0.024	0.016^f^ (0.003)	0.011 to 0.021

a TARI: Traffic Accident Risk Index.

b ref: reference group.

c *P*<.05.

d SES: socioeconomic status.

e *P*<.01.

f *P*<.001.

### Factors Associated With TARI and the 3 Subdomains Among MFDWs

The results of the multiple linear regression analyses for TARI and its 3 subdomains, including near-miss experiences, other-rated accident anxiety, and self-rated accident anxiety, are presented in Table 3. All 4 regression models were statistically significant, with adjusted *R*^2^ values of 29.9%, 21.6%, 29.2%, and 23.5% for the TARI, near-miss experiences, other-rated accident anxiety, and self-rated accident anxiety models, respectively. Multicollinearity was assessed across all regression models, and all variance inflation factors were less than 10, indicating no significant multicollinearity. When TARI was the dependent variable, the statistically significant factors included break time, helmet use, physical activity, and occupational fatigue. Compared with participants with break times of 2 hours or more, those with break times of 30 minutes to 1 hour had lower perceived traffic accident risk (*B*=−0.329, 95% CI −0.650 to −0.007; *P*=.045). Additionally, inconsistent helmet use (*B*=0.413, 95% CI 0.030 to 0.796; *P*=.04), lack of physical activity (*B*=0.368, 95% CI 0.016 to 0.719; *P*=.04), and higher levels of occupational fatigue (*B*=0.017, 95% CI 0.012 to 0.021; *P*<.001) were associated with higher TARI scores.

In the model with near-miss experiences as the dependent variable, the statistically significant factors included break times, smoking, and occupational fatigue. Compared with participants with break times of 2 hours or more, those with break times of less than 30 minutes (*B*=−0.474, 95% CI −0.936 to −0.011; *P*=.045) or between 30 minutes and 1 hour (*B*=−0.470, 95% CI −0.846 to −0.095; *P*=.01) were associated with lower levels of near-miss experiences. Additionally, smoking (*B*=0.298, 95% CI 0.041 to 0.555; *P*=.02) and higher levels of occupational fatigue (*B*=0.016, 95% CI 0.011 to 0.021; *P*<.001) were associated with increased near-miss experiences.

In the model with other-rated accident anxiety as the dependent variable, statistically significant factors included educational level, helmet use, and occupational fatigue. Participants with an educational level of middle school or lower (*B*=0.525, 95% CI 0.036 to 1.014; *P*=.04), compared with those with a college education or higher, those with inconsistent helmet use (*B*=0.495, 95% CI 0.025 to 0.966; *P*=.04), and those with higher occupational fatigue (*B*=0.019, 95% CI 0.013 to 0.024; *P*<.001) were associated with greater other-rated anxiety about accidents.

Finally, in the model with self-rated accident anxiety as the dependent variable, statistically significant factors included helmet use, physical activity, and occupational fatigue. Participants with inconsistent helmet use (*B*=0.493, 95% CI 0.036 to 0.949; *P*=.04), those who did not engage in physical activity (B=0.586, 95% CI 0.168 to 1.005; *P*=.006) compared with those who exercised almost daily, and those with higher levels of occupational fatigue (*B*=0.016, 95% CI 0.011 to 0.021; *P*<.001) were associated with higher self-rated accident anxiety.

## Discussion

This study contributes to the growing body of literature on MFDWs by adopting a multidimensional view of traffic safety beyond crash-based outcomes. We examined TARI, near-miss experiences, and 2 forms of accident-related anxiety (self-rated and other-rated anxiety). This distinction is meaningful because behavioral precursors to accidents (near-miss experiences) and the psychological dimensions of safety (accident anxiety) may not share identical correlates. By modeling these outcomes separately, our findings offer a more granular understanding of how occupational fatigue and health behaviors are related to safety precursors and perceived vulnerability in platform-based delivery work.

Across all the models, occupational fatigue emerged as the most consistent correlate of adverse safety outcomes. Higher fatigue was associated with a higher overall traffic accident risk, more near-miss experiences, and greater accident-related anxiety. This pattern is broadly consistent with prior evidence indicating that fatigue is linked to degraded cognitive performance and safety-critical decision-making [20,21]. Additionally, fatigue has been associated with reduced situational awareness and slower reaction times, which have been linked to near-miss events and judgment errors in complex traffic environments [22,23]. The consistency of the association across multiple safety domains suggests that fatigue is a salient marker of increased safety risks and psychological strain in MFDWs. Although causal inference was not warranted in this cross-sectional study, the consistency of fatigue associations across all safety domains suggests that fatigue management warrants prioritization in this workforce. At the platform level, immediate steps could include in-app advisory alerts recommending rest after extended delivery periods, as well as the integration of fatigue-related considerations into the delivery system design, followed by rider-facing educational interventions that raise awareness of fatigue-inducing factors and promote monitoring. Looking further ahead, the development of cumulative fatigue monitoring systems integrated into delivery platforms, as well as policy-level requirements for platforms to disclose algorithmic workload indicators to occupational health authorities, should be pursued.

Several modifiable health behaviors were independently associated with specific safety domains, even after adjusting for fatigue and other covariates. The inconsistent use of helmets was associated with a higher overall accident risk and greater levels of self-rated and other-rated accident anxiety, consistent with the evidence that helmet use remains a key protective factor for motorcyclists [10,24]. Insufficient physical activity was associated with higher overall risk and self-rated accident anxiety. This finding aligns with broader evidence linking physical activity to lower anxiety symptoms and improved mental well-being [25] and may plausibly relate to self-regulation or coping capacity relevant to safety, although the underlying mechanisms in this occupational context remain unclear and warrant further investigation. Smoking was specifically associated with higher near-miss experiences, which may reflect distraction-related or broader risk-prone patterns [11,26-28]. Together, these findings provide preliminary evidence supporting a multicomponent view of safety promotion, suggesting that fatigue management may be most effective when considered alongside targeted health-behavior strategies.

The multivariable models explained a significant proportion of the variance in the outcomes (adjusted *R*²=29.9% for TARI, 21.6% for near-miss experiences, 29.2% for other-rated accident anxiety, and 23.5% for self-rated accident anxiety), suggesting that fatigue and the included behavioral and occupational covariates capture important correlates of multidimensional safety. At the same time, the remaining unexplained variance indicates that additional work- and context-related determinants may also contribute to safety outcomes and should be incorporated in future studies.

We also observed a counterintuitive inverse association between longer self-reported break duration and safety outcomes, with riders reporting breaks of 2 hours or more showing higher accident risk and near-miss experiences. This counterintuitive finding should not be interpreted as evidence that shorter breaks are protective or that reducing rest improves safety among MFDWs. This finding should be regarded as exploratory and hypothesis generating, as alternative explanations cannot be excluded, including reverse causation and potential sensitivity to how break duration was categorized. One plausible hypothesis raised by this study is that the observed pattern may reflect a structural measurement limitation inherent in algorithmically managed delivery. In platform-based food delivery, periods that MFDWs label as “breaks” may frequently coincide with unpaid on-duty waiting time, during which riders remain logged into the app and available for dispatch [5]. If so, those reporting the longest “breaks” may not represent a well-rested MFDW, but rather one with prolonged on-duty availability that sustains cognitive load rather than enabling genuine recovery. Recovery research suggests that the restorative value of rest depends not only on its duration but also on psychological detachment from work demands [29], and such detachment may be structurally difficult to achieve within algorithmically managed gig work. Previous studies further suggested that prolonged idle periods can induce compensatory time pressure once orders resume, which may be associated with riskier riding behavior [4,30]. However, these explanations remain speculative, and this hypothesis requires direct empirical testing in future studies. Future studies should employ more precise measures that distinguish restorative rest from on-duty waiting, ideally complemented by objective indicators such as platform activity logs or ecological momentary assessments.

This study had some limitations. First, its cross-sectional design precluded causal inferences. However, given that MFDWs represent a particularly vulnerable occupational group in which traffic incidents frequently result in serious injuries [3], identifying strong correlates such as occupational fatigue provides critical and immediate evidence for prioritizing interventions, even in the absence of longitudinal confirmation. Second, self-reported measures may have introduced recall or reporting bias. In particular, break times may not clearly distinguish actual rest from idle waiting times. Nevertheless, it is important to note that psychological constructs, such as “accident anxiety” and “near-miss experiences,” are inherently subjective. These internal vulnerability states cannot be captured through objective accident records alone, making self-reporting an appropriate and valid assessment method. As the 2 primary psychological constructs, occupational fatigue and TARI, were assessed using a single self-report survey, the possibility of common method bias cannot be fully excluded. To examine this issue, we conducted Harman single-factor test, which showed that the first factor accounted for 45.78% of the total variance, below the commonly used 50% threshold. Third, several important unmeasured variables may limit the interpretation of our findings. Objective exposure indicators such as riding distance, number of deliveries per shift, shift timing, route type, urban traffic exposure, weather conditions, incentive pressure, and platform algorithm demands were not captured. Given that these factors may vary considerably across MFDWs and could independently influence safety outcomes, the findings regarding TARI and its subdomains should be interpreted with caution. Furthermore, residual confounding cannot be ruled out, as health behaviors such as smoking and physical inactivity may partly reflect unmeasured risk-prone tendencies, and unmeasured work-context factors may have contributed to the observed associations. Finally, because convenience sampling was employed, the findings should be generalized with caution. In particular, South Korea’s food delivery market is characterized by a predominance of algorithmic dispatch systems, peak-hour demand concentration, and highly developed road infrastructure, which may differ substantially from delivery contexts in other countries [31,32]. Therefore, findings should be interpreted with caution when applied to other national settings. Consequently, future studies should use longitudinal designs and more precise measurements of rest and workload, along with objective exposure indicators, to validate these findings. Real-time approaches, such as ecological momentary assessments and complementary objective indicators, may help clarify temporal relationships and support more actionable guidance on fatigue management and recovery for MFDWs working under algorithmically managed piece-rate conditions.

This study contributes to the literature on MFDWs by conceptualizing traffic safety as a multidimensional construct encompassing accident risk, near-miss experiences, and accident-related anxiety. Occupational fatigue was consistently associated with all the outcomes, suggesting its utility as an important indicator of elevated safety risks and accident-related anxiety. Modifiable health behaviors, including inconsistent helmet use, insufficient physical activity, and smoking, were also associated with specific safety domains, indicating the potential value of considering fatigue management alongside health-behavior approaches. The inverse association observed between longer self-reported break duration and safety outcomes warrants cautious interpretation, as break time may partly reflect on-duty waiting rather than restorative rest. Future studies using longitudinal designs and more precise measures of rest and exposure are required to clarify temporality and inform context-sensitive strategies for supporting recovery during platform delivery.

## Supplementary material

10.2196/92667 Multimedia Appendix 1 Exploratory univariate comparisons of the Traffic Accident Risk Index and its three domains by participants’ characteristics (N=336).

10.2196/92667 Multimedia Appendix 2 Pearson correlations between traffic accident risk outcomes and occupational fatigue among motorcycle-based food delivery workers (N=336).

10.2196/92667 Checklist 1 STROBE checklist.

## Abbreviations

MFDW: motorcycle-based food delivery worker
STROBE: Strengthening the Reporting of Observational Studies in Epidemiology
TARI: Traffic Accident Risk Index
