# Supplementary material for: Occupational Fatigue and Multidimensional Traffic Risk Outcomes Among Motorcycle-Based Food Delivery Workers: Cross-Sectional Study
Source: JMIR Public Health Surveill. 2026 Jun 12;12:e92667. doi: 10.2196/92667 (PMC13263021; doi:10.2196/92667)
Supplement: Multimedia Appendix 1 [file publichealth-v12-e92667-s001.docx]

Multimedia appendix 1. Exploratory univariate comparisons of the TARI^a^ and its three domains by MFDWs’ characteristics (*N* = 336)

| Variables | Characteristics | TARI | | Near Miss Experience | | Other-rated accident anxiety | | Self-rated accident anxiety | |
| --- | --- | --- | --- | --- | --- | --- | --- | --- | --- |
|  |  | M ± SD | t or F | M ± SD | t or F | M ± SD | t or F | M ± SD | t or F |
| Demographics | Age^†^ |  |  |  |  |  |  |  |  |
|  | 18–29 ^a^ | 2.52 ± 0.95 | 3.43^*^ | 2.86 ± 1.10 | 3.73^*^ | 2.46 ± 1.13 | 2.69 | 2.25 ± 1.04 | 2.87 |
|  | 30–39 ^b^ | 2.81 ± 0.94 | (a < b) | 3.14 ± 0.98 |  | 2.74 ± 1.29 |  | 2.55 ± 1.17 |  |
|  | ≥ 40 ^c^ | 2.68 ± 1.02 |  | 3.29 ± 1.16 |  | 2.34 ± 1.11 |  | 2.40 ± 1.18 |  |
|  | Gender |  |  |  |  |  |  |  |  |
|  | Female | 2.41 ± 0.94 | -1.52 | 3.02 ± 1.13 | -0.08 | 2.13 ± 1.16 | -2.17^*^ | 2.09 ± 1.04 | -1.55 |
|  | Male | 2.68 ± 0.97 |  | 3.03 ± 1.08 |  | 2.61 ± 1.20 |  | 2.41 ± 1.12 |  |
|  | Marital status |  |  |  |  |  |  |  |  |
|  | Single | 2.62 ± 0.97 | 1.23 | 2.94 ± 1.06 | 3.96^*^ | 2.57 ± 1.22 | 0.34 | 2.34 ± 1.09 | 1.28 |
|  | Married | 2.83 ± 1.01 |  | 3.31 ± 1.13 | (a < b) | 2.60 ± 1.17 |  | 2.58 ± 1.22 |  |
|  | Divorced/widowed | 2.65 ± 0.62 |  | 3.46 ± 0.99 |  | 2.29 ± 1.12 |  | 2.21 ± 0.86 |  |
|  | Education^†^ |  |  |  |  |  |  |  |  |
|  | ≤ Middle school ^a^ | 3.18 ± 0.91 | 9.96^***^ | 3.23 ± 1.05 | 2.67 | 3.34 ± 1.34 | 14.61^***^ | 2.96 ± 1.24 | 9.53^***^ |
|  | High school ^b^ | 2.54 ± 0.97 | (b, c < a) | 2.90 ± 1.07 |  | 2.42 ± 1.12 | (b, c < a) | 2.30 ± 1.08 | (b, c < a) |
|  | ≥ College ^c^ | 2.59 ± 0.92 |  | 3.14 ± 1.10 |  | 2.40 ± 1.12 |  | 2.22 ± 1.00 |  |
|  | Social economic status^†^ |  |  |  |  |  |  |  |  |
|  | High ^a^ | 2.94 ± 0.92 | 5.76^**^ | 3.14 ± 1.27 | 4.30^*^ | 3.11 ± 1.27 | 5.15^**^ | 2.56 ± 1.16 | 3.76^*^ |
|  | Moderate ^b^ | 2.50 ± 0.93 | (b < c) | 2.88 ± 1.06 | (b < c) | 2.39 ± 1.15 | (b < a) | 2.23 ± 1.02 | (b < c) |
|  | Low ^c^ | 2.84 ± 0.99 |  | 3.23 ± 1.06 |  | 2.73 ± 1.23 |  | 2.57 ± 1.21 |  |
| Occupational status | Career year^†^ |  |  |  |  |  |  |  |  |
|  | 1 year or less ^a^ | 2.36 ± 1.01 | 6.09^***^ | 2.89 ± 1.28 | 3.47^*^ | 2.06 ± 1.02 | 8.35^***^ | 2.12 ± 1.04 | 3.15^*^ |
|  | Over 1 year to 3 years ^b^ | 2.53 ± 0.96 | (a, b < c) | 2.87 ± 1.06 | (b < c) | 2.42 ± 1.18 | (a, b, d < c) | 2.28 ± 1.09 | (a, b < c) |
|  | Over 3 years to 5 years ^c^ | 2.97 ± 0.89 |  | 3.26 ± 0.96 |  | 3.00 ± 1.18 |  | 2.64 ± 1.13 |  |
|  | 5 years or more ^d^ | 2.62 ± 1.06 |  | 3.25 ± 1.29 |  | 2.27 ± 1.23 |  | 2.33 ± 1.18 |  |
|  | Working hours per week^†^ |  |  |  |  |  |  |  |  |
|  | < 40 ^a^ | 2.83 ± 0.94 | 5.76^**^ | 3.10 ± 1.06 | 6.43^**^ | 2.73 ± 1.33 | 2.95 | 2.66 ± 1.18 | 5.63^**^ |
|  | 40–52 ^b^ | 2.45 ± 1.00 | (b < a, c) | 2.79 ± 1.11 | (b < c) | 2.38 ± 1.16 |  | 2.18 ± 1.07 | (b < a) |
|  | > 52 ^c^ | 2.79 ± 0.91 |  | 3.29 ± 0.99 |  | 2.66 ± 1.11 |  | 2.41 ± 1.06 |  |
|  | Break including mealtime^†^ |  |  |  |  |  |  |  |  |
|  | ≤ 30 mins ^a^ | 2.93 ± 1.01 | 4.19^**^ | 3.11 ± 1.07 | 3.09^*^ | 2.97 ± 1.41 | 5.01^**^ | 2.72 ± 1.29 | 3.63^*^ |
|  | 30 mins to 1 hr ^b^ | 2.49 ± 0.93 | (b < a) | 2.84 ± 1.03 | (b < d) | 2.36 ± 1.11 | (b, c < a) | 2.27 ± 1.01 | (b, c < a) |
|  | 1 hr to 2 hrs ^c^ | 2.58 ± 0.96 |  | 3.07 ± 1.15 |  | 2.43 ± 1.12 |  | 2.23 ± 1.05 |  |
|  | ≥ 2 hrs ^d^ | 2.80 ± 0.91 |  | 3.41 ± 1.04 |  | 2.63 ± 1.02 |  | 2.37 ± 1.06 |  |
|  | Helmet use |  |  |  |  |  |  |  |  |
|  | Yes | 2.61 ± 0.96 | -3.29^**^ | 2.99 ± 1.09 | -2.48^*^ | 2.51 ± 1.21 | -3.09^**^ | 2.34 ± 1.10 | -2.78^**^ |
|  | No | 3.29 ± 0.79 |  | 3.57 ± 0.82 |  | 3.30 ± 0.93 |  | 3.00 ± 1.11 |  |
| Health-related | BMI |  |  |  |  |  |  |  |  |
| characteristics | Underweight to normal | 2.77 ± 0.93 | 2.11^*^ | 3.07 ± 1.07 | 0.70 | 2.72 ± 1.20 | 2.43^*^ | 2.52 ± 1.06 | 2.19^*^ |
|  | Overweight to obese | 2.55 ± 0.99 |  | 2.98 ± 1.09 |  | 2.40 ± 1.20 |  | 2.25 ± 1.16 |  |
|  | Smoking |  |  |  |  |  |  |  |  |
|  | Current smoker | 2.63 ± 0.92 | -0.43 | 3.26 ± 1.07 | 3.05^**^ | 2.37 ± 1.12 | -2.24^*^ | 2.25 ± 1.05 | -1.64 |
|  | Non-smoker | 2.68 ± 1.00 |  | 2.89 ± 1.07 |  | 2.67 ± 1.24 |  | 2.46 ± 1.14 |  |
|  | Alcohol problem |  |  |  |  |  |  |  |  |
|  | Yes | 2.85 ± 0.88 | -2.21^*^ | 2.93 ± 1.10 | -2.75^**^ | 2.51 ± 1.23 | -1.38 | 2.33 ± 1.11 | -1.59 |
|  | No | 2.59 ± 0.99 |  | 3.30 ± 0.99 |  | 2.71 ± 1.15 |  | 2.54 ± 1.10 |  |
|  | Physical activity |  |  |  |  |  |  |  |  |
|  | Almost every day ^a^ | 2.67 ± 0.88 | 3.00^*^ | 2.91 ± 1.11 | 1.26 | 2.81 ± 1.37 | 3.28^*^ | 2.29 ± 1.05 | 3.68^*^ |
|  | 3–4 times/week ^b^ | 2.70 ± 0.89 | (c < d) | 3.08 ± 0.98 |  | 2.69 ± 1.15 |  | 2.33 ± 0.98 | (c < d) |
|  | 1–2 times/week ^c^ | 2.41 ± 0.95 |  | 2.87 ± 1.13 |  | 2.24 ± 1.09 |  | 2.13 ± 1.00 |  |
|  | Almost never ^d^ | 2.80 ± 1.01 |  | 3.13 ± 1.08 |  | 2.65 ± 1.24 |  | 2.60 ± 1.22 |  |
|  | Sleep duration |  |  |  |  |  |  |  |  |
|  | < 7 hrs or > 9 hrs | 2.71 ± 1.00 | -0.89 | 3.10 ± 1.14 | -0.99 | 2.57 ± 1.27 | -0.35 | 2.46 ± 1.17 | -1.01 |
|  | 7 to 9 hrs | 2.61 ± 0.96 |  | 2.98 ± 1.05 |  | 2.52 ± 1.15 |  | 2.33 ± 1.07 |  |
|  | Presence of disease |  |  |  |  |  |  |  |  |
|  | Yes | 2.93 ± 0.94 | -2.20^*^ | 3.44 ± 1.05 | -2.89^**^ | 2.83 ± 1.20 | -1.66 | 2.55 ± 1.01 | -1.14 |
|  | No | 2.61 ± 0.97 |  | 2.96 ± 1.07 |  | 2.52 ± 1.21 |  | 2.36 ± 1.13 |  |

Notes. Values are presented as mean (SD). Between-group differences were assessed using independent t-test for binary variables and on-way analysis if variance (ANOVA) for variables with three air more categories. All-p-value are two-sided and reported for exploratory purposes with no adjustment for multiple comparisons. Post-hoc pairwise comparisons were conducted using Bonferroni correction. TARI, the Traffic Accident Risk Index; M, mean; SD, standard deviation; BMI, body mass index. ^†^Post-hoc test was performed using Bonferroni correction.

^a^TARI: Traffic Accident Risk Index.

^b^MFDWs: motorcycle-based food delivery workers.
* *p* < .05, ** *p* < .01, *** *p* < .001
