# Supplementary material for: Occupational Fatigue and Multidimensional Traffic Risk Outcomes Among Motorcycle-Based Food Delivery Workers: Cross-Sectional Study
Source: JMIR Public Health Surveill. 2026 Jun 12;12:e92667. doi: 10.2196/92667 (PMC13263021; doi:10.2196/92667)
Supplement: Multimedia Appendix 2 [file publichealth-v12-e92667-s002.docx]

Multimedia appendix 2. Person correlations between traffic accident risk outcomes and occupational fatigue among MFDWs (*N* = 336)

| Variables | r (*p*) | | | | |
| --- | --- | --- | --- | --- | --- |
|  | 1 | 2 | 3 | 4 | 5 |
| 1. TARI | - |  |  |  |  |
| 2. Near-miss experiences | .77  (<.001) | - |  |  |  |
| 3. Other-rated accident anxiety | .90  (<.001) | .50  (<.001) | - |  |  |
| 4. Self-rated accident anxiety | .89  (<.001) | .50  (<.001) | .77  (<.001) | - |  |
| 5. Occupational fatigue | .52  (<.001) | .36  (<.001) | .51  (<.001) | .46  (<.001) | - |

Notes: Near-miss experiences, other-rated accidental anxiety, self-rated accident anxiety are subscales of the TARI measure. MFDWs, motorcycle-based food delivery workers; TARI, the Traffic Accident Risk Index.
